# Editorial: Cellular Mechanisms of Aging and Longevity in Oral Health and Disease

**DOI:** 10.3389/froh.2022.971191

**Published:** 2022-07-12

**Authors:** Christopher W. Cutler, Gill Diamond

**Affiliations:** ^1^Department of Periodontics, The Dental College of Georgia-Augusta University, Augusta, GA, United States; ^2^Department of Oral Immunology and Infectious Diseases, University of Louisville, Louisville, KY, United States

**Keywords:** periodontitis, Alzheimer's disease, senescence, inflammaging, microbiome

The aging adult population will continue to grow well into the next two decades [1] with a rise expected in diseases of inflammaging, as reviewed by Clark et al. Particular emphasis in this review is placed on periodontitis (PD), one of the most common age-related inflammatory disease [2]. PD is comorbid with many inflammaging diseases such as type 2 diabetes, heart disease [3], cancer [4], and Alzheimer's disease [5]. Collectively these comorbid diseases constitute a major cause of mortality and morbidity on the globe [3–6]. Intense efforts are needed to identify the pathogenic mechanisms involved, and to facilitate the development of novel therapeutic agents. COVID 19 deaths have also been linked to advanced age [7], with human [8] and murine studies [9] beginning to reveal the destructive inflammatory lung responses [10]. Similar studies are defining the destructive cellular immune responses in PD [11], with particular emphasis on *in situ* studies in humans [12] and in mice [13], documenting an important role for unregulated activation of gingival dendritic cells and T cells *in situ* in promotion of Th17 driven alveolar bone loss. Understanding how these immune cells interact with the oral microbiome in young and aged subjects and promote systemic dissemination of oral pathogens [3, 12, 14] is of particular significance. Ebersole et al. examined the age-related changes of innate antimicrobial factors at oral mucosa and secretions in non-human primates subjected to experimental PD. Antimicrobial factors in the oral environment must battle microbes such as the keystone periodontal pathogen *Porphyromonas gingivalis* [15]. This species has been discovered in the brains of Alzheimer's disease patients [16], and invades dendritic cells, resulting in activation of the senescence associated secretory phenotype (SASP). The SASP releases a burst of exosomes into the milieu, promoting senescence in normal bystander immune cells [17]. Parkinson and Prime have provided a Mini-review of classical cellular senescence and its implications for oral tumor surveillance and therapeutics, thus rounding out this topical section.

## Author Contributions

CC wrote the text. GD edited the text. All authors contributed to the article and approved the submitted version.

## Conflict of Interest

The authors declare that the research was conducted in the absence of any commercial or financial relationships that could be construed as a potential conflict of interest.

## Publisher's Note

All claims expressed in this article are solely those of the authors and do not necessarily represent those of their affiliated organizations, or those of the publisher, the editors and the reviewers. Any product that may be evaluated in this article, or claim that may be made by its manufacturer, is not guaranteed or endorsed by the publisher.
